# Triglyceride–glucose index (TyG index) is a predictor of incident colorectal cancer: a population-based longitudinal study

**DOI:** 10.1186/s12902-020-00581-w

**Published:** 2020-07-24

**Authors:** Takuro Okamura, Yoshitaka Hashimoto, Masahide Hamaguchi, Akihiro Obora, Takao Kojima, Michiaki Fukui

**Affiliations:** 1 Department of Endocrinology and Metabolism, Kyoto Prefectural University of Medicine, Graduate School of Medical Science, 465, Kajii-cho, Kawaramachi-Hirokoji, Kamigyo-ku, Kyoto, 602-8566 Japan; 2 Department of Gastroenterology, Asahi University Hospital, Gifu, Japan

**Keywords:** Cohort study, Colorectal cancer, TyG index, Insulin resistance, Epidemiology

## Abstract

**Background:**

Colorectal cancer (CRC), which is related with insulin resistance, is a one of the most common cancers. Triglyceride-glucose index (TyG index) was made for a marker of insulin resistance. We conducted the investigation of association between TyG index and incident CRC.

**Methods:**

We examined the affect of TyG index on incident CRC in this historical cohort study of 27,944 (16,454 men and 11,490 women) participants. TyG index was calculated as ln [fasting triglycerides (mg/dL) × fasting plasma glucose (mg/dL)/2]. The impact of TyG index on incident CRC was investigated using Cox proportional hazard models, adjusting for sex, age, body mass index, smoking status, alcohol consumption, exercise, systolic blood pressure and creatinine. The covariate-adjusted receiver operating characteristic (ROC) curve calculated the area under the curve (AUC) and cut-off value of TyG index for the incidence of CRC.

**Results:**

During the median 4.4-year follow-up, 116 participants were diagnosed as CRC. The cumulative incidence rate of CRC were 0.4%. In Cox proportional hazard model, the HRs of TyG index were 1.38 (95% Confidence interval (CI), 1.00–1.91, *p* = 0.049) after adjusting for covariates. In the covariate-adjusted ROC curve analysis, the cut-off value of TyG index for incident CRC was 8.272 (AUC 0.687 (95%CI, 0.637–737, sensitivity = 0.620, specificity = 0.668, *p* < 0.001)).

**Conclusions:**

TyG index can predict the onset of CRC. For early detection of CRC, we should encourage people with high TyG index to undergo screening for CRC.

## Background

Colorectal cancer (CRC) is the third leading cause of the most common cancers next to lung and breast cancer in the world [1]. The most efficient strategies in reducing incident CRC are identification of patients with high-risk for CRC and performing colonoscopy.

Metabolic syndrome, i.e. obesity, impaired glucose tolerance, hypertension and dyslipidemia, has been reported to be associated with risk of CRC by several studies [2–6]. Particularly, insulin resistance is associated with hyperinsulinemia [7, 8], increased levels of insulin-like growth factors (IGF) [9, 10], and alterations in necrosis factor (NF)-κB [11] and peroxisome proliferator-activated receptor gamma (PPARγ) signaling [12], which plays a key role in the pathogenesis of CRC [13–15].

Triglyceride-glucose index (TyG index) was made for a marker of insulin resistance, and calculated with fasting plasma glucose and triglycerides [16, 17]. .In addition, several groups demonstrated that TyG index could predict the incidence of type 2 diabetes [18, 19] and cardiovascular disease [20]. Thus, we hypothesized that TyG index can predict the incidence of CRC. However, no previous studies have investigated this association. We conducted the investigation of association between TyG index and incident CRC in this retrospective study.

## Methods

### Study participants and study design

This was a retrospective sub-analysis of the ongoing cohort study named NAGALA (NAfld in the Gifu Area, Longitudinal Analysis) study, which is a medical checkup program and a cohort investigation that has been ongoing at Asahi University Hospital (Gifu, Japan). The impact of TyG index on the risk of incident CRC was investigated in this NAGALA study. This investigation has been ongoing and aims to detect chronic diseases and their risk factors, and to promote public health. The detail of NAGALA study and medical examination programs were expressed in our previous study [21]. We recruited the participants in this medical examination program from 2003 to 2016. The exclusion criteria were follows: the participants whose data, including smoking status and alcohol consumption, were missed. The informed consent to participate was provided from all patients in this sub-analysis of NAGALA study. The present study was approved by each hospital’s Ethics Committee.

### Data collection and measurements

In a standardized self-administered questionnaire, the medical background and the data of alcohol consumption, smoking status and physical activity were taken from all of the participants [21]. In the questionnaire, we asked about the type and amount of alcohol consumption per week during the prior month, then we estimated the mean ethanol intake per week. The participants were categorized into the following four groups: no or minimal alcohol consumer, < 40 g/week; light, 40–140 g/week; moderate, 140–280 g/week; or heavy alcohol consumer, > 280 g/week [22]. In addition, the participants were categorized into the following three groups by smoking status: non-, ex-, or current smoker. Furthermore, we also asked participants’ recreational activities and sports activities by questionnaire. We defined regular exercisers as individuals regularly over one time per week playing any type of sports [23]. Lastly, TyG index was calculated as ln [fasting triglycerides (mg/dL) × fasting plasma glucose (mg/dL)/2] [16].

### Identification of cases of colorectal cancer

We performed fecal occult blood test in this health checkup program. When the fecal occult test was positive and the presence of CRC was suspected, the staffs informed and recommended participants to receive further examinations such as lower gastrointestinal endoscopy. We gathered the medical information regarding colorectal cancers by a standardized letter from a hospital where a participant receives further examinations. Besides, we included participants who received further examinations for reasons other than occult blood test and were diagnosed as colorectal cancer. The gastroenterologist checked the gathered information and defined them as colorectal cancer. From Jan 1st 2004, we adopted the standardized letter. Then, we set the study period as Jan 1st 2004 to Dec 31st 2016. The primary endpoint in this study was set as incident CRC. In this study, we defined the day when participants were suggested as cancer at the health checkup center as the onset day if participants were diagnosed as CRC.

### Statistical analysis

We performed the following analyses with the JMP software ver. 13.0 (SAS, Cary, NC, USA). A *p*-value was considered to indicate a significant when it was less than 0.05. We expressed values as mean (SD) or median (interquartile) for continuous variables and number (%) for categorial variables. The baseline characteristics of participants who developed CRC and those who did not were investigated. The *p* values were calculated uby one-way analysis of variance (ANOVA) for continuous variables and chi-squared test for categorical variables, respectively.

Cox proportional hazards models were used to estimate hazard ratio (HR) and 95% confidence interval (CI) for incident CRC. In Model 1, we unadjusted, in Model 2, we adjusted for sex and age, and, in Model 3, we adjusted for Model 2 and body mass index, smoking status, alcohol consumption, exercise habit, systolic blood pressure and serum creatinine.

Additionally, the covariate-adjusted receiver operating characteristic (ROC) curve calculated the area under the curve (AUC) and cut-off value of TyG index for the incidence of CRC, and the AUC of TyG index was compared with those of BMI, brinkman index, alcohol consumption, total cholesterol, and triglycerides.

## Results

**Fig. 1:**
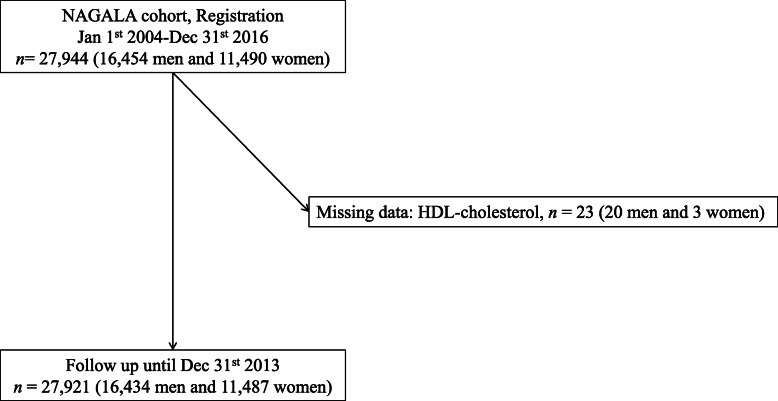
Study flow diagram for the registration of participants

From Jan 1st in 2004 to Dec 31st in 2016, 27,944 participants (16,454 men and 11,490 women) were registered in NAGALA cohort. After excluding those who met exclusion criteria, 27,921 participants (16,434 men and 11,487 women) were entered (Fig. 1). The baseline characteristics of the participants are shown in Table 1. Mean (± standard deviation) age of participants was 45.7 ± 10.1 years old and BMI was 22.6 ± 3.3 kg/m^2^.

**Table 1:** Clinical characteristics of participants

	**Total (*n*** **= 27,921)**
Sex, men/women	16,434/11,487
Age, yrs	45.7 (10.1)
BMI, kg/m^2^	22.6 (3.3)
WC, cm	78.1 (9.6)
Ex-smoker	6185 (22.2)
Current-smoker	6921 (24.9)
Regular exerciser	4984 (18.0)
Light alcohol consumer	2984 (10.8)
Moderate alcohol consumer	2577 (9.3)
Heavy alcohol consumer	2198 (8.0)
Fasting plasma glucose, mmol/L	5.3 (0.7)
HbA1c, %	5.3 (0.6)
HbA1c, mmol/mol	34.6 (5.4)
Triglycerides, mmol/L	1.0 (0.9)
Total cholesterol, mmol/L	5.2 (0.9)
HDL cholesterol, mmol/L	1.4 (0.4)
AST, IU/L	19.5 (9.7)
ALT, IU/L	21.5 (15.2)
GGT, IU/L	23.6 (25.3)
SBP, mmHg	117.7 (16.4)
DBP, mmHg	73.6 (11.2)
Creatinine, Creatinine, μmol/L	73.0 (22.8)
TyG index	8.2 (0.7)

*ALT* alanine transaminase, *AST* aspartate transaminase, *BMI* body mass index, *DBP* diastolic blood pressure, *GGT* γ-glutamyltransferase, *HbA1c* hemoglobin A1c, *HDL* high density lipoprotein, *SBP* systolic blood pressure, *TyG index* triglyceride–glucose index, *WC* waist circumference

Data are mean (SD) or number

Participants who developed CRC were older than those who did not (51.1 ± 9.3 vs 45.6 ± 10.1 years, *p* < 0.001). Next, alcohol consumption of participants who developed CRC was greater than that of those who did not. In the metabolic status, fasting plasma glucose, hemoglobin A1c, triglycerides, total cholesterol, serum creatinine, and systolic and diastolic blood pressure of participants who developed CRC were higher than those of participants who did not (Table 2).

**Table 2:** Clinical characteristics of participants

	**CRC (−)** **(*n*** **= 27,805)**	**CRC (+)** **(*n*** **= 116)**	***P*-value**
Sex, men/women	16,353/11,452	81/35	0.014
Age, yrs	45.6 (10.1)	51.1 (9.3)	< 0.001
BMI, kg/m^2^	22.6 (3.3)	23.1 (3.5)	0.084
WC, cm	78.0 (9.6)	80.5 (8.9)	0.006
Smoking status	–	–	0.003
Non-smoker	14,772 (53.0)	43 (37.1)	–
Ex-smoker	6150 (22.1)	35 (30.2)	–
Current smoker	6883 (24.8)	38 (32.8)	–
Regular exerciser	4964 (18.0)	20 (17.5)	0.905
Alcohol consumption	–	–	< 0.001
Light alcohol consumer	4682 (17.0)	23 (20.0)	–
Moderate alcohol consumer	2560 (9.3)	17 (14.8)	–
Heavy alcohol consumer	2177 (7.9)	21 (18.3)	–
Fasting plasma glucose, mmol/L	5.4 (1.0)	5.7 (1.4)	0.004
HbA1c, %	5.3 (0.6)	5.4 (0.8)	0.042
HbA1c, mmol/mol	34.6 (6.8)	35.9 (8.2)	0.042
Triglycerides, mmol/L	1.0 (0.9)	1.3 (1.0)	0.002
Total cholesterol, mmol/L	5.2 (0.9)	5.4 (0.9)	0.011
HDL cholesterol, mmol/L	1.4 (0.4)	1.3 (0.4)	0.012
Creatinine, μmol/L	73.0 (22.8)	76.7 (16.1)	0.039
AST, IU/L	19.5 (9.7)	19.1 (7.6)	0.664
ALT, IU/L	21.5 (15.2)	20.9 (9.2)	0.689
GGT, IU/L	23.6 (25.2)	28.1 (32.3)	0.053
SBP, mmHg	117.7 (16.4)	121.2 (17.1)	0.018
DBP, mmHg	73.6 (11.2)	76.7 (11.0)	0.004
TyG index	8.2 (0.7)	8.4 (0.7)	< 0.001

*ALT* alanine transaminase, *AST* aspartate transaminase, *BMI* body mass index, *CRC* colorectal cancer, *DBP* diastolic blood pressure, *GGT* γ-glutamyltransferase, *HbA1c* hemoglobin A1c, *HDL* high density lipoprotein, *SBP* systolic blood pressure, *TyG index* triglyceride–glucose index, WC: waist circumference

Data are mean (SD) or number

**Fig. 2:**
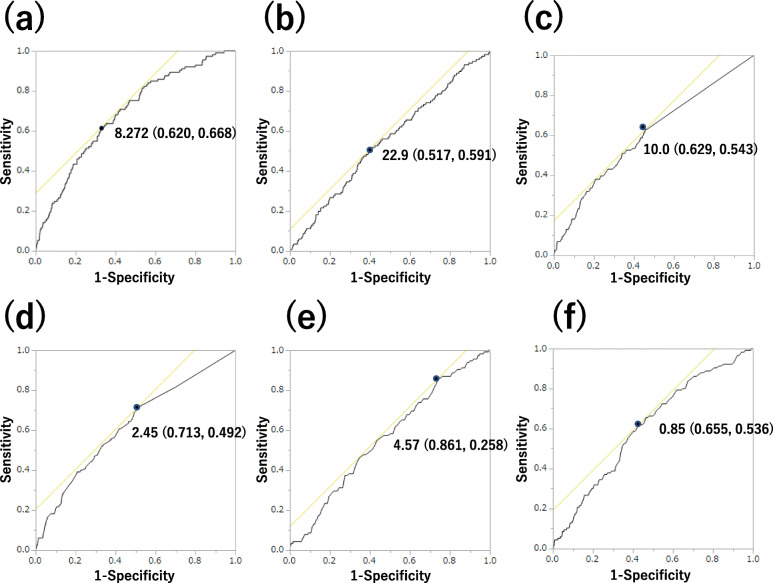
Area under the covariate-adjusted receiver operating characteristic curve (AUC) for predicting incident colorectal cancer (CRC). **a**.TyG index, **b**. BMI, **c**. brinkman index, **d**. alcohol consumption, **e**. TC, f. TG. The optimal cut-off value of TyG index for incident CRC is 8.272 (AUC 0.687 (95%CI, 0.637–737, sensitivity = 0.620, specificity = 0.668, *p* < 0.001)). AUC of BMI was 0.547 (95% CI: 0.498–0.602, *p* < 0.001, vs. TyG index), that of brinkman index was 0.605 (95% CI: 0.551–0.654, *p* < 0.001, vs. TyG index), that of alcohol consumption was 0.623 (95% CI: 0.571–0.676, *p* < 0.001, vs. TyG index), that of TC was 0.563 (95% CI: 0.515–0.616, *p* < 0.001, vs. TyG index), and that of TG was 0.608 (95% CI: 0.560–0.658, *p* < 0.001, vs. TyG index), respectively

One hundred sixteen participants were diagnosed as CRC during the median 4.4-year follow-up. The cumulative incidence rate of CRC was 0.4%. In Cox proportional hazard model, the HRs of TyG index were 1.65 (95%CI 1.29–2.13, *p* < 0.001) in Model 1, 1.41 (1.05–1.89, *p* = 0.021) in Model 2 and 1.36 (1.00–1.86, *p* = 0.049) in Model 3 (Table 3 qaxzdf). Moreover, the cut-off value of TyG index for incident CRC was 8.272 in the covariate-adjusted ROC curve analysis (AUC 0.687 (95%CI, 0.637–0.737, sensitivity = 0.620, specificity = 0.668, *p* < 0.001)). Additionally, we also calculated AUC of BMI (0.547, 95% CI: 0.498–0.602, *p* < 0.001, vs. TyG index), that of brinkman index (0.605, 95% CI: 0.551–0.654, *p* < 0.001, vs. TyG index), that of alcohol consumption (0.623, 95% CI: 0.571–0.676, *p* < 0.001, vs. TyG index), that of TC (0.563, 95% CI: 0.515–0.616, *p* < 0.001, vs. TyG index), and that of TG (0.608, 95% CI: 0.560–0.658, *p* < 0.001, vs. TyG index), respectively (Fig. 2). TyG index had a significantly higher AUC for the development of CRC than the other risk factors.

**Table 3:** Cox proportional hazards for incident colorectal cancer

	**Model 1**		**Model 2**		**Model 3**	
	**HR (95% CI)**	***p*-value**	**HR (95% CI)**	***p*-value**	**HR (95% CI)**	***p*-value**
Men	–	–	1.09 (0.71–1.70)	0.712	1.01 (0.59–1.84)	0.975
Age, years	–	–	1.07 (1.05–1.09)	< 0.001	1.07 (1.04–1.09)	< 0.001
BMI, kg/m^2^	–	–	–	–	0.99 (0.93–1.07)	0.912
Ex-smoker	–	–	–	–	1.48 (0.84–2.61)	0.176
Current smoker	–	–	–	–	1.55 (0.87–2.75)	0.117
Light alcohol consumer	–	–	–	–	1.32 (0.77–2.24)	0.298
Moderate alcohol consumer	–	–	–	–	1.55 (0.87–2.75)	0.137
Heavy alcohol consumer	–	–	–	–	2.02 (1.14–3.57)	0.016
Regular exerciser	–	–	–	–	0.79 (0.46–1.29)	0.360
Systolic blood pressure, mmHg	–	–	–	–	0.99 (0.99–1.01)	0.987
Creatinine, μmol/L	–	–	–	–	0.85 (0.25–2.89)	0.794
TyG index	1.65 (1.29–2.13)	< 0.001	1.41 (1.05–1.89)	0.021	1.38 (1.00–1.91)	0.049

*BMI* body mass index, *TyG index* triglyceride-glucose index. Exercise was defined as nonregular exerciser (0) or regular exerciser (1), smoking status was defined as nonsmoker (0), ex-smoker (1) or current smoker (2), and alcohol consumption was defined as no or minimal alcohol consumer (0), light alcohol consumer (1), moderate alcohol consumer (2) and heavy alcohol consumer (3). Model 1 was unadjusted. Model 2 was adjusted for sex and age. Model 3 was adjusted for sex, age, body mass index, smoking status, alcohol consumption, exercise, systolic blood pressure and serum creatinine

## Discussion

In this retrospective cohort study of over 27,000 Japanese participants, we investigated the affect of TyG index on incidence of CRC and, for the first time, demonstrated that TyG index could predict incidence of CRC. TyG index, calculated with fasting triglycerides and plasma glucose, was originally proposed as a biomarker of insulin resistance [16, 17], and some following studies demonstrated TyG index could predict the incidence of type 2 diabetes [18, 19] and cardiovascular disease [20]. In addition, we showed that the value of TyG index of 8.272 was a cut-off value for incident of CRC. In fact, we compared area under the curve (AUC) of TyG index with that of the known risk factors of the CRC: BMI, smoking, alcohol consumption [24], TC, and TG [25]. Then, TyG index was superior to other markers.

Several human studies previously reported the association between insulin resistance and risk of colorectal cancer [13–15]. Similarly, several basic researches revealed that insulin resistance was reported to be associated with hyperinsulinemia [7, 8], increased levels of IGF [9, 10], and alterations in NF-κB [11] and PPARγ signaling [12], which may play a key role in the pathogenesis of CRC.

Insulin resistance induces hyperinsulinemia, which activates PI3K/Akt/mTOR/S6K signaling pathway in cancer [26]. Additionally, elevated serum TG, a component of the TyG index, activates Akt signaling pathway through G protein-coupled receptor [27]. In fact, the gene expression of the PI3K/Akt/mTOR signaling axis in human colorectal cancer are significantly overexpressed compared to normal colonic tissues [28]. Moreover, some recent studies reported that insulin promotes colon cancer progression by upregulation of acyl coenzyme A: cholesterol acyltransferase1 [29], and increases the expression of vascular cell adhesion molecule-1 in intestinal tumor endothelial cells and produces proinflammatory state which promotes tumorigenesis [30]. Taken together, hyperinsulinemia caused by insulin resistance may contribute to the growth and progression of colorectal cancer.

Insulin resistance is related with increased IGF-1 levels [9, 10]. IGF-1 activates IGF-1 receptor, which regulate cell proliferation, survival, and angiogenesis [31]. IGF-1 promotes to product vascular endothelial growth factor, which supports tumor growth [32]. In fact, the expression of IGF-1 and IGF-1 receptor increases with tumor size in colorectal cancer in a human study [33]. In addition, hyperglycemia enhances the cellular sensitivity to IGF-I, including increased cell proliferation and migration [34].

Hyperglycemia is reported to induce an increase in intranuclear NF-κB in human subjects [35]. NF-κB regulates a number of genes included in the process of cell proliferation, neoplasia, and metastasis. Moreover, two target genes for NF-κB such as cyclin D1 and cMyc take an important part in cell growth and proliferation [36]. In the carcinogenic process of CRC, the NF-κB signaling pathway is an important complex pathway. The changes of gene expression in signaling pathway by insulin resistance downregulated the genes with a positive role of immune response and upregulated the genes of pro-inflammatory, which induced potent immune response and inflammation [37].

Sarraf, et al. [38] demonstrated that the growth and differentiation of colon cancer cells can be modulated through PPARγ, and since then, many groups have reported the association between PPARγ signaling and CRC [39–41]. Kubota, et al. [42] demonstrated the association between PPARγ and insulin resistance in adipocyte. In addition, colorectal tumor is developed by the activation of PPARγ [43].

In addition, both serum plasma glucose and triglycerides levels are related with risk of CRC in human subjects [2, 6]. TyG index is composed of levels of triglyceride and glucose, and it is expected that TyG index is more useful risk marker of CRC.

Our study also has some limitations. We did not perform colonoscopy to all participants. In addition, the proportion of the participants who received further examinations was approximately only 50% out of the participants whose fecal occult tests were positive. Therefore, there was a possibility that the number of incident CRC was underestimated. However, CRC incidence in this study was 0.4% and that available for all generations in Japan is 0.2% [44]. Therefore, underestimation of incident CRC might be smaller than expected. Lastly, in the present study, only the TyG index at the initial visit was included in the analysis, and we did not take into account factors of improvement and deterioration during the course of follow-up.

## Conclusions

In conclusion, TyG index is a useful and accessible tool for predicting incident CRC. For early detection of CRC, we should encourage people with high TyG index, the cut-off value of which was 8.272 in our study, to undergo screening for CRC.

## Data Availability

The datasets used and analysed during the current study available from the corresponding author on reasonable request.

## Abbreviations

ANOVA: Analysis of variance
AUC: Area under the curve
CI: Confidence interval
CRC: Colorectal cancer
HR: Hazard ratio
IGF: Insulin-like growth factors
NF: Necrosis factor
PPARγ: Peroxisome proliferator-activated receptor gamma
ROC: Receiver operating characteristic
TyG index: Triglyceride-glucose index
